# Supplementary material for: Patterns of Intron Gain and Loss in Fungi
Source: PLoS Biol. 2004 Nov 30;2(12):e422. doi: 10.1371/journal.pbio.0020422 (PMC532390; doi:10.1371/journal.pbio.0020422)
Supplement: Table S1 — Also available at http://genes.mit.edu/NielsenEtAl/. (4.3 MB ZIP). [file pbio.0020422.st001.zip › NielsenEtAl/html/462.html]

AN8016.1.NCU01234.1.MG04885.1.FG05858.1


```
 CLUSTAL W (1.82) Multiple Sequence Alignments - Introns Inserted


Sequence 1: MG04885.1	401 aa
Sequence 2: FG05858.1	401 aa
Sequence 3: NCU01234.1	400 aa
Sequence 4: AN8016.1	399 aa
Alignment Length: 401 aa
Number Identitical Residues: 370 aa
Alignment Score (without introns) 14209


MG04885.1 	MAEGSGIDRKAEE~RMEFTTSKEVTVHPTFESMALKE1SLLRGIYAYGYESPSAVQSRAI
NCU01234.1	MADG-GIDRKADE~KIQFSTSKEVTVHPTFESMSLKE1SLLRGIYAYGYESPSAVQSRAI
FG05858.1 	MAEGSGIDRKADE~RMEFSTSKEVTVHPTFESMSLKE1NLLRGIYAYGYESPSAVQSRAI
AN8016.1  	MADG--IDRRADD1KMEFNTSKEVTVAPTFEDMHLKE~SLLRGIYAYGYESPSAVQSRAI
          	**:*  ***:*:: :::*.******* ****.* *** .*********************

MG04885.1 	VQVCKGRDTIAQAQSGTGKTATFSISMLQVIDTAVRETQA~LVLSPTRELATQIQSVVMA
NCU01234.1	VQICKGRDTIAQAQSGTGKTATFSISMLQVIDTAVRETQA~LVLSPTRELATQIQSVVMA
FG05858.1 	VQVCKGRDTIAQAQSGTGKTATFSISMLQVIDTAVRETQA~LVLSPTRELATQIQSVVMA
AN8016.1  	VQICKGRDTIAQAQSGTGKTATFSISALQVIDTVVRETQA1LVLSPTRELATQIQSVIMA
          	**:*********************** ******.****** ****************:**

MG04885.1 	LGDYMNVQCHACIGGTNVGEDIRKLDYGQHIVSGTPGRVADMIRRRHLRTRHIKMLVLDE
NCU01234.1	LGDYMNVQCHACIGGTNVGEDIRKLDYGQHIVSGTPGRVADMIRRRHLRTRHIKMLVLDE
FG05858.1 	LGDYMNVQCHACIGGTNVGEDIRKLDYGQHIVSGTPGRVADMIRRRHLRTRHIKMLVLDE
AN8016.1  	LGDYMNVQCHACIGGTNIGEDIRKLDYGQHVVSGTPGRVADMIRRRHLRTRHIKMLVLDE
          	*****************:************:*****************************

MG04885.1 	ADELLNQGFREQIYDVYRYLPPATQVVVVSATLPYDVLDMTTKFMTDPVRILVKRDELTL
NCU01234.1	ADELLNQGFREQIYDVYRYLPPATQVVVVSATLPYDVLDMTTKFMTDPVRILVKRDELTL
FG05858.1 	ADELLNKGFREQIYDVYRYLPPATQVVVVSATLPYDVLDMTTKFMTDPVRILVKRDELTL
AN8016.1  	ADELLNRGFREQIYDVYRYLPPATQVVVVSATLPYDVLDMTTKFMTDPVRVLVKRDELTL
          	******:*******************************************:*********

MG04885.1 	EGLKQYFIAIEKEDWKFDTLCDLYDTLTITQAVIFCNTRRKVDWLTDKMREANFTVSSMH
NCU01234.1	EGLKQYFIAVEKEDWKFDTLCDLYDTLTITQAVIFCNTRRKVDWLTDKMREANFTVSSMH
FG05858.1 	EGLKQYFIAVEKEDWKFDTLCDLYDTLTITQAVIFCNTRRKVDWLTDKMREANFTVSSMH
AN8016.1  	EGIKQYFIAVEKEEWKFDTLCDLYDTLTITQAVIFCNTRRKVDWLTDKMREANFTVSSMH
          	**:******:***:**********************************************

MG04885.1 	GDMPQKERDSIMQDFRQGNSRVLISTDVWARGIDVQQVSLVINYDLPSNRENYIHRIGRS
NCU01234.1	GDMPQKERDSIMQDFRQGNSRVLISTDVWARGIDVQQVSLVINYDLPSNRENYIHRIGRS
FG05858.1 	GDMPQKERDSIMQDFRQGNSRVLISTDVWARGIDVQQVSLVINYDLPSNRENYIHRIGRS
AN8016.1  	GEMPQKERDSIMQDFRQGNSRVLISTDVWARGIDVQQVSLVINYDLPTNRENYIHRIGRS
          	*:*********************************************:************

MG04885.1 	GRFGRKGVAINFVTSEDVRILRDIEL1YYSTQIDEMPMNV~ADLIA
NCU01234.1	GRFGRKGVAINFVTSEDVRILRDIEL1YYSTQIDEMPMNV~ADLIS
FG05858.1 	GRFGRKGVAINFVTTEDVRILRDIEL1YYSTQIDEMPMNV~ADLIA
AN8016.1  	GRFGRKGVAINFVTSDDVRILRDIEL~YYSTQIDEMPMNV1ADLLS
          	**************::********** ************* ***::
```
